# Human Gut Microbiota Changes Reveal the Progression of Glucose Intolerance

**DOI:** 10.1371/journal.pone.0071108

**Published:** 2013-08-27

**Authors:** Xiuying Zhang, Dongqian Shen, Zhiwei Fang, Zhuye Jie, Xinmin Qiu, Chunfang Zhang, Yingli Chen, Linong Ji

**Affiliations:** 1 Department of Endocrinology and Metabolism, Peking University People's Hospital, Peking University Diabetes Centre, Beijing, China; 2 BGI-Shenzhen, Shenzhen, China; 3 Department of Clinical Epidemiology, Peking University People's Hospital, Beijing, China; University of Tor Vergata, Italy

## Abstract

To explore the relationship of gut microbiota with the development of type 2 diabetes (T2DM), we analyzed 121 subjects who were divided into 3 groups based on their glucose intolerance status: normal glucose tolerance (NGT; n = 44), prediabetes (Pre-DM; n = 64), or newly diagnosed T2DM (n = 13). Gut microbiota characterizations were determined with 16S rDNA-based high-throughput sequencing. T2DM-related dysbiosis was observed, including the separation of microbial communities and a change of alpha diversity between the different glucose intolerance statuses. To assess the correlation between metabolic parameters and microbiota diversity, clinical characteristics were also measured and a significant association between metabolic parameters (FPG, CRP) and gut microbiota was found. In addition, a total of 28 operational taxonomic units (OTUs) were found to be related to T2DM status by the Kruskal-Wallis H test, most of which were enriched in the T2DM group. Butyrate-producing bacteria (e.g. *Akkermansia muciniphila* ATCCBAA-835, and *Faecalibacterium prausnitzii* L2-6) had a higher abundance in the NGT group than in the pre-DM group. At genus level, the abundance of *Bacteroides* in the T2DM group was only half that of the NGT and Pre-DM groups. Previously reported T2DM-related markers were also compared with the data in this study, and some inconsistencies were noted. We found that *Verrucomicrobiae* may be a potential marker of T2DM as it had a significantly lower abundance in both the pre-DM and T2DM groups. In conclusion, this research provides further evidence of the structural modulation of gut microbiota in the pathogenesis of diabetes.

## Introduction

Type 2 diabetes mellitus (T2DM) has become one of the fastest growing public health problems in both developed and developing countries. In China, for instance, the prevalence of diabetes and prediabetes have reached 9.7% and 15.5%, respectively, and account for 92.4 million adults and 148.2 million adults, respectively, according to the National Diabetes and Metabolic Disorders Study conducted from 2007 to 2008 [1]. In many countries, type 2 diabetes mellitus has now become the most prevalent type of diabetes in children [2]. Prediabetes is even more prevalent than type 2 diabetes and there is an important demarcation line between the two conditions that indicates whether an individual is going to develop diabetes and cardiovascular disease [1], [3]. Therefore, a more comprehensive understanding of the dynamic development processes of glucose intolerance is fundamentally important for understanding the mechanisms responsible for the development of T2DM and thus strategies to prevent it.

T2DM is thought to involve a complex process involving genetic susceptibility and environmental factors, both of which remain only partially understood. Genome-wide association strategies have offered great opportunities for investigation of the pathogenesis of the disease. However, common genetic variants that are associated with diabetes have only very slightly improved the prediction of future T2DM [4], [5]. Growing evidence that multiple environmental factors contribute to their development was exemplified by emerging evidence of the role of the intestinal microbiota as a potential novel contributor to this epidemic. The metagenomic sequencing of the human microbiome has revealed that there are 3.3 million non-redundant genes, with over 99% of the genes being of bacterial origin [6]. This gene set contains at least100-fold more genes than the complete human genome. With >50 species shared by 90% of the individuals studied, considerable variation occurs in both the types of microbes and in the diversity of microbial functional genes between individuals [6]–[8].

Recent research has shown that gut bacteria play an important role in disorders such as obesity, diabetes, and cardiovascular diseases. Compositional changes of human gut microbiota in response to weight status have been examined in many studies. Gordon and colleagues have reported that human obesity is associated with a low abundance of intestinal *Bacteroidetes* and a high abundance of *Firmicutes*, in agreement with results from a weight-loss program involving consumption of restricted diets for a year [9]–[11]. However, Duncan et al. [12] and Jumpertz et al. [13] found no difference in the proportions of fecal microbiota between lean and obese subjects. Relatively few studies have reported the relationship between the gut microbiota composition and T2DM. Larsen et al. [14] showed that men with T2DM had significantly reduced levels of fecal *Firmicutes*, including *Clostridia*, compared with non-diabetic control subjects, and that the ratio of *Bacteroidetes* to *Firmicutes*, as well as the ratio of *Bacteroides*-*Prevotella* to *C. coccoides*-*E. rectale*, was correlated positively with the plasma glucose concentration but not with body mass index (BMI). In order to characterize the gut microbiota in diabetic patients, Wu et al. [15] analyzed the diversity and similarity of gut microbiota in 16 T2DM patients and 12 healthy individuals and found that genus *Bifidobacterium* and *Bacteroides vulgatus* were less represented in the microbiota of the diabetic group than the non-diabetic group. In our recent metagenome-wide association study of Chinese T2DM patients, we found that patients withT2DM were characterized by a moderate degree of gut microbial dysbiosis, a decrease in the abundance of some universal butyrate-producing bacteria, and an increase in various opportunistic pathogens [16].

Recent population-based studies conducted to evaluate the association between gut microbiota and diabetes have produced conflicting results. Were they limited by the relatively low number of subjects, the diversity of patient enrollment, technical issues, or by racial differences? Probably, bacterial sequences specific for T2DM act as signatures of glucose tolerance status rather than obesity. Moreover, a comprehensive understanding of the microbiota characteristics associated with the natural course of T2DM also remains to be achieved.

In this study, we hypothesized that the proportion and diversity of intestinal microbiota in humans are changed before the period of prediabetes. The aims of the study were to explore the relationships between gut microbiota and different glucose intolerance statuses in a Chinese Han population using 16S rDNA-based high-throughput sequencing.

## Results

### Clinical characteristics

The anthropometric and metabolic characteristics of the 3 study groups are shown in Table 1. Body mass index (BMI), waist-hip ratio (WHR), fasting plasma glucose (FPG), plasma glucose 2 hours after oral glucose challenge (2HPG), triglyceride (TG), fasting insulin (FINS) concentration, 2-hour insulin (2HINS) concentration, insulin resistance index (IR), and C-reactive protein (CRP) values were all significantly higher in the T2DM group than in the NGT group (*P*<0.05). In addition, BMI, WHR, FPG, 2HPG, FINS, 2HINS, IR, and CRP values were also higher in the Pre-DM group than in the NGT group (*P*<0.05). However, there were no significant differences in LDL-C and HDL-C levels between the 3 groups.

**Table 1:** Characteristics of the subjects with NGT, prediabetes and T2DM.

Variable	NGT(n = 44)	Pre-DM(n = 64)	T2DM(n = 13)
Age (years)	55 (9)	54 (7)	52 (9)
BMI (kg/m^2^)	23.38 (8.62)	24.95 (7.18)*	26.50 (8.61)†
Waist-hip ratio (WHR)	0.85 (3.52)	0.88 (2.96)*	0.90 (3.47)†
SBP (mmHg)	122 (15)	126 (13)*	129 (11)
DBP (mmHg)	77 (8)	81 (9)*	86 (9)†
FPG (mmol/L)^a^	5.28 (4.91, 5.51)	5.74 (5.28, 6.29)*	7.36 (7.09, 8.21)† ^,^ ‡
2HPG (mmol/L)^a^	6.81 (6.19, 7.45)	9.58 (8.61, 10.92)*	13.52 (12.41, 14.19)† ^,^ ‡
FINS (mIU/L)^a^	6.6 (4.84, 9.21)	9.83 (7.1, 13.86)*	19.15 (11.22, 25.32)† ^,^ ‡
2HINS (mIU/L)^a^	35.87 (26.93, 57.03)	76.2 (53.77, 127.9)*	80.27 (47.5, 114.1)†
IR^a^	1.53 (1.08, 2.39)	2.63 (1.73, 3.67)*	6.29 (3.69, 8.04)† ^,^ ‡
TG (mmol/L)^a^	1.35 (0.9, 1.69)	1.41 (1.04, 1.74)	1.84 (1.6,0 2.58)† ^,^ ‡
TC (mmol/L^a^	5.0 (4.25, 5.44)	4.88 (4.47, 5.54)	4.89 (4.67, 5.47)
HDL-C (mmol/L)^a^	1.29 (1.02, 1.53)	1.19 (1.09, 1.32)	1.12 (0.94, 1.2)
LDL-C (mmol/L)^a^	2.95 (2.5, 3.54)	3.10 (2.77, 3.74)	3.0 (2.69, 3.13)
CRP (mg/L)^a^	1.02 (0.44, 2.07)	1.58 (1.03, 2.51)*	2.12 (1.38, 3.27)†

a Median (interquartile range).

* *P*<0.05 for Pre-DM vs NGT;

† *P*<0.05 for T2DM vs NGT;

‡ *P*<0.05 for T2DM vs Pre-DM.

BMI = body mass index; CPR = C-reactive protein; DBP = diastolic blood pressure; FINS = fasting insulin; FPG = fasting plasma glucose; HDL-C = high-density lipoprotein cholesterol; 2HINS = 2-hour insulin concentration; 2HPG = 2-hour plasma glucose concentration; IR = insulin resistance index; LDL-C = low-density lipoprotein cholesterol; NGT = normal glucose tolerance; Pre-DM = prediabetes; SBP = systolic blood pressure; TC = total cholesterol; T2DM = type 2 diabetes mellitus; TG = triglyceride.

### Characteristics of gut microbiota at different glucose intolerance levels

A total of 2.2 million sequence reads were generated from the 16S rDNA gene V3–V5 amplicons, with an average of 9474 (±3470 SD) reads per subject. To obtain a detailed structural overview of the microbiome of each subject enrolled in the study, operational taxonomic unit (OTU) analysis was conducted and yielded a total of 8,107 OTUs with a 97% similarity cutoff. *Firmicutes* and *Bacteroidetes* were found to be the dominant taxa of the overall structures of the microbiome at phylum level, as were *Clostridia* and *Bacteroidia* at class level. However, *Clostridia* had a higher abundance in the T2DM group compared with the NGT and Pre-DM groups. At genus level, a total of 159 genera were identified but only 31 genera had an abundance more than 0.1% in at least 1 of the 3 groups. The relative abundance of *Bacteroides* in the T2DM group (∼10%) was lower than that in the NGT and Pre-DM groups, while *Dorea*, *Prevotella* and *Collinsella* had higher relative abundances in the T2DM group than in the NGT group.

Interestingly, the abundance of *Streptococcus* continued to decrease from the NGT group to the Pre-DM group, and to the T2DM group. Additionally, the average relative abundances of genus *Prevotella* and genus *Megamonas* in the Pre-DM group were 1.89-fold and 8.83-fold greater, respectively, than in the NGT group. All of these results indicate that dysbiosis of the gut microbial structure had occurred. To fully explore the relationships between microbes and metabolic disorders, a profile at genus level was generated to perform future analyses, including reads that can only be assigned to a family level. The 20 most abundant clades were then determined (Figure S1).

#### Analysis of the similarity and diversity of microbial communities among different glucose tolerance statuses

**Figure 1:**
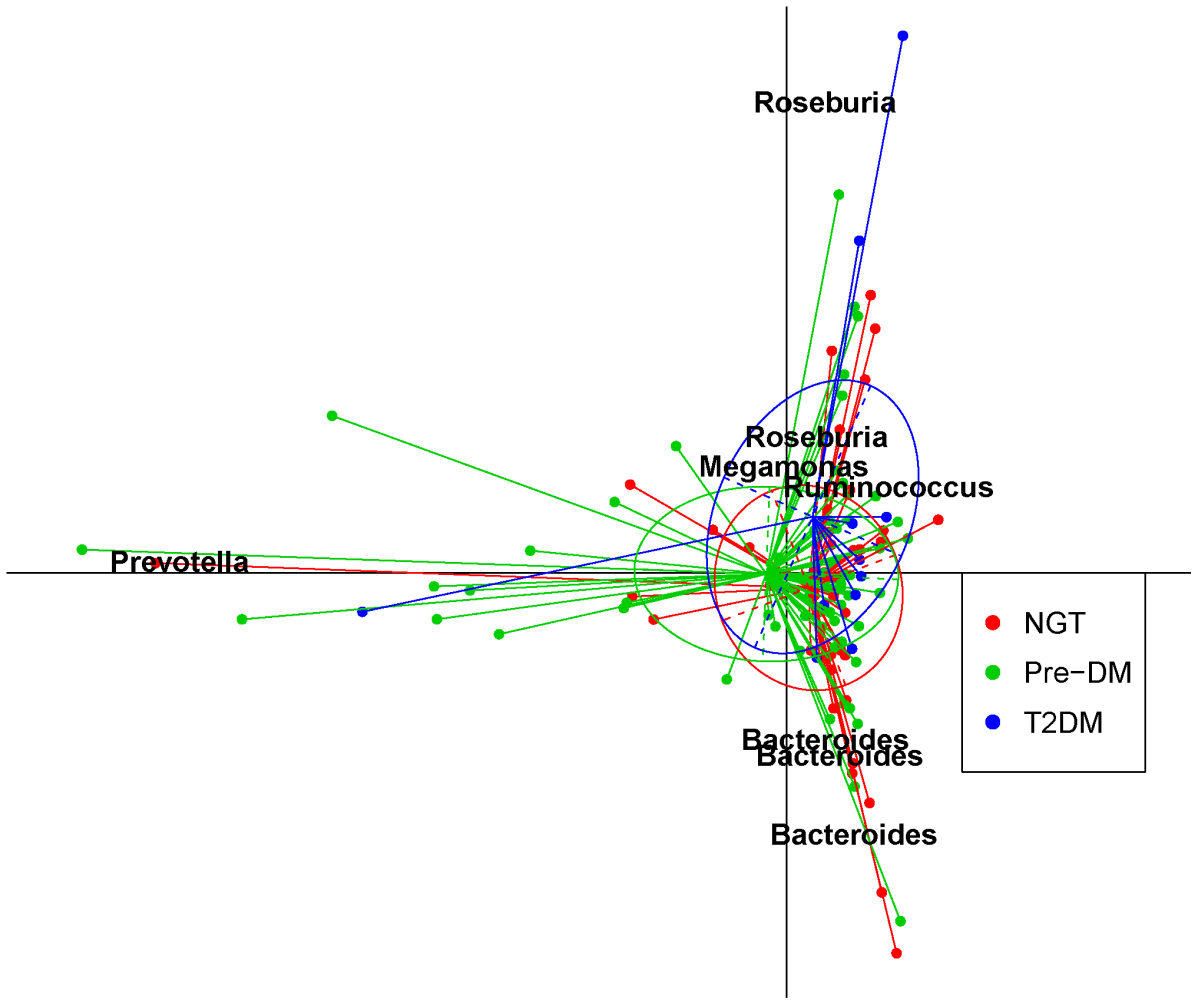
Principal component analysis (PCA) analysis of the similarity of microbiota (OTUs) between the NGT, Pre-DM, and T2DM groups. Data for NGT (n = 44), Pre-DM (n = 64) and T2DM (n = 13) subjects were plotted on the first two principal components of the OTU profiles. The first 2 components (contributing 19.5% of variance) were plotted. Lines connect individuals belonging to the same group and colored circles cover individuals near the center of gravity for each cluster (<1.5*σ*). The top 7 OTUs (labeled with their annotations) for the main contributors to these groups were determined and plotted by their loadings for these 2 components. PCA was performed with R package ‘ade4’. OTU = operational taxonomic unit.

Principal component analysis (PCA) was employed to assess the similarity of microbial communities between the 3 groups. PCA results based on 8,107 OTUs showed a slight separation between the NGT and T2DM groups (Figure 1), which was also observed by PCA based on the taxonomic profile (Figure S2). However, the PERMANOVA test, which was used to assess changes in the overall structure, indicated no notable separation among the 3 groups (*P* = 0.2626) (Table S1).

**Figure 2:**
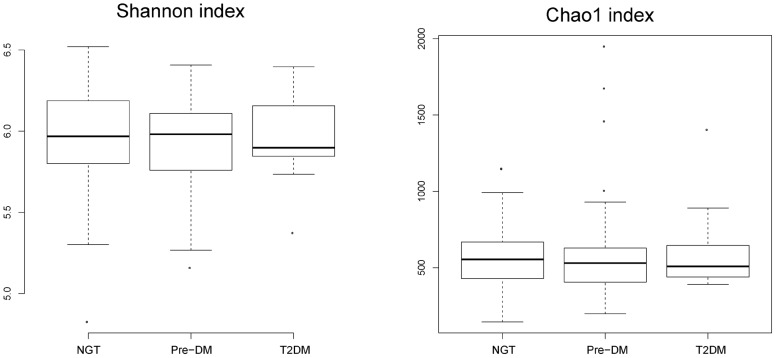
Alpha diversity of the 3 groups. The Shannon index (left panel) and Chao1 index (right panel) were computed for all 121 subjects. The box depicts the interquartile range (IQR) between the first and third quartiles (25th and 75th percentiles, respectively) and the line inside the box denotes the median.

The Chao1 index and Shannon index were calculated to estimate the alpha diversity. No significant differences were found between the 3 groups, but T2DM subjects showed a slightly decreased diversity compared with the NGT group (Figure 2). Using the method documented in the Meta-HIT study [17], we identified 3 enterotypes that were mainly enriched in *Bacteroides*, *Prevotella* and *Ruminococcus*, respectively. Interestingly, this was not associated with T2DM status, and the Fisher exact test was not significant (*P* = 0.355; Figure S3).

#### OTUs differentially enriched in the NGT and T2DM groups

When we examined a total of 1,640 OTUs that occurred in more than 6 samples, 28 OTUs were found to be associated with a different glucose intolerance status by the Kruskal-Wallis H test (Table 2 and Figure S4; first error rate 1%). Table 2 shows the complete list of OTUs that exhibited different abundances between the 3 groups. OTU4416 (*Subdoligranulum*) had a relatively higher abundance in the T2DM group than in the NGT and Pre-DM groups, and the average abundance of OTU286 (*Clostridiales*) in T2DM subjects was twice that in NGT subjects. Seven OTUs annotated to *Lachnospiraceae* were enriched in the T2DM group compared with the NGT group, and all 3 OTUs annotated to *Ruminococcus* were enriched in the T2DM group compared with the NGT group. Additionally, OTU5579, OTU2980, OTU7046 and OTU6565 annotated to *Eubacterium*, *Sporobacter*, *Abiotrophia* and *Peptostreptococcus*, respectively, were enriched significantly in the T2DM group compared with the NGT group, especially OTU7046, the mean abundance of which was 20 times that in the NGT group.

**Table 2:** Kruskal-Wallis H tests of associations between glucose intolerance status and microbiota composition (OTUs level).

OTU	*P*	Occurrence rate			Taxon	Identity
		NGT	Pre-DM	T2DM		
**NGT enrichment:**						
6275	0.0081626	0.18	0.02	0.08	*Bacteroides*	1
2101	0.0064251	0.77	0.56	0.38	*Blautia*	0.98
2340	0.0087904	0.07	0.25	0.00	*Clostridiales*	1
3265	0.0054547	0.27	0.52	0.08	*Haemophilus*	0.86
4375	0.001644	0.32	0.59	0.23	*Lachnospiraceae*	0.99
1632	0.009626	0.11	0.30	0.00	*Megamonas*	1
3209^a^	0.0024805	0.16	0.39	0.00	*Megamonas*	1
1900	0.003299	0.50	0.22	0.46	*Roseburia*	1
**T2DM enrichment:**						
7678	0.0037128	0.05	0.31	0.23	*Bacteroides*	1
770	0.0057936	0.02	0.23	0.08	*Bacteroides*	1
6679	0.0047138	0.84	0.63	1.00	*Blautia*	1
286	0.0020471	0.14	0.05	0.38	*Clostridiales*	1
5579	0.0071113	0.32	0.13	0.46	*Eubacterium*	0.96
396	0.0010595	0.11	0.42	0.15	*Lachnospiraceae*	0.81
2508	0.0009765	0.36	0.70	0.54	*Lachnospiraceae*	0.84
3325	0.0067516	0.30	0.56	0.62	*Lachnospiraceae*	0.87
6803	0.0017342	0.55	0.83	0.69	*Lachnospiraceae*	0.87
6718^b^	0.0031246	0.39	0.56	0.85	*Lachnospiraceae*	0.95
4235	0.0098852	0.20	0.50	0.38	*Lachnospiraceae*	0.96
3249	0.00129	0.07	0.06	0.38	*Lachnospiraceae*	1
6565	0.0062553	0.07	0.09	0.38	*Peptostreptococcus*	1
7853	0.0005223	0.07	0.05	0.38	*Ruminococcaceae*	0.97
469	0.0022403	0.14	0.13	0.54	*Ruminococcus*	0.97
4393	0.004243	0.18	0.14	0.54	*Ruminococcus*	1
4508^b^	0.0028564	0.39	0.48	0.85	*Ruminococcus*	1
2980	0.0039087	0.05	0.11	0.38	*Sporobacter*	0.92
4416	0.0045742	0.50	0.39	0.85	*Subdoligranulum*	0.97
7046	0.0024899	0.02	0.06	0.31	*Abiotrophia*	0.97

a The relative abundance of OTUs enriched in the NGT group decreased to 0 with the development of T2DM.

b The relative abundance of OTUs increased with the development of T2DM.

NGT = normal glucose tolerance; OTU = operational taxonomic unit; Pre-DM = prediabetes; T2DM = type 2 diabetes mellitus.

*Haemophilus* (OTU3265) and *Megamonas* (OTU1632 and OTU3209) showed high relative abundances in the NGT group compared with the T2DM group. Moreover, *Roseburia* (OTU1900) showed a notably high relative abundance in NGT subjects compared with the other groups.

### Association between microbiota diversity and clinical indicators

#### PERMANOVA tests of associations between microbiota composition and clinical parameters

The PERMANOVA test was used to test the overall effect of gut microbiota on healthy parameters. The model was adjusted for BMI, age and sex as these might be confounding factors. The results showed that fasting plasma glucose (FPG) was significantly associated with the gut microbiota (*P* = 0.0453) in all samples. In the NGT group, the CRP concentration was significantly associated with microbiota (Table 3).

**Table 3:** PERMANOVA tests of associations between clinical parameters and microbiota composition (adjusted for age, gender and BMI).

Phenotype	Df	Sum of squares	Mean squares	F model	R^2^	Pr (>F)
**All groups (95 samples):**						
FPG	1	0.451807	0.451807	1.35295	0.01423	0.0453
**NGT group (33 samples):**						
CRP	1	0.431633	0.431633	1.320166	0.040869	0.055
**T2DM group (12 samples):**						
FPG	1	0.428187	0.428187	1.324532	0.113103	0.0605

BMI = body mass index; CRP = C-reactive protein; FPG = fasting plasma glucose; PERMANOVA = permutational multivariate analysis of variance.

#### Co-inertia analysis (CIA) of microbiota at the OTU level and clinical parameters

**Figure 3:**
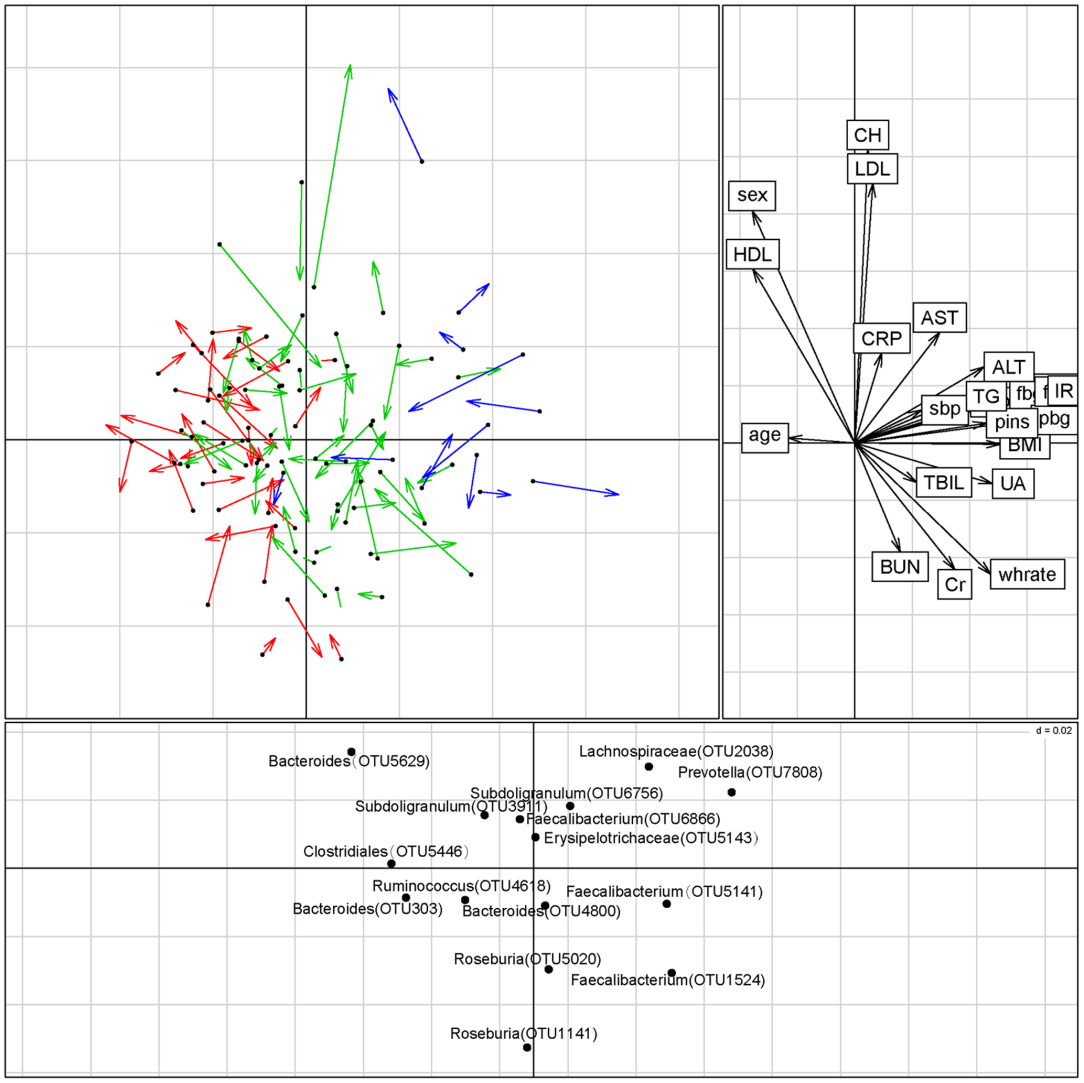
Co-inertia analysis (CIA) of the relationship between microbiota at the OTU level, clinical parameters, and disease group. The upper left panel shows the CIA of the clinical parameter principal component analysis (PCA) and the microbiota PCA; arrows indicate where samples in the clinical parameter dataset are relative to the microbiota dataset. Red lines represent the NGT group, green lines the Pre-DM group, and blue lines the T2DM group. The upper right panel shows clinical parameter loading data; the lower panel displays the associated microbiota at the OTU level (labeled with their annotations). Only OTUs present in at least 1% of the samples were used in the analysis. CIA was performed with R package ‘ade4’. OTU = operational taxonomic unit.

CIA of OTU abundance and clinical parameters revealed a modest relationship (RV coefficient = 0.237, permutation 1000 times, *P* value = 0.196) between the two datasets (Figure 3). The major genus dedicating to separation was consistent with the Kruskal-Wallis test result referred to above.

The first two components of the CIA accounted for 47.3% of the variance in the datasets, with component 1 (horizontal) accounting for 31.1% of the variance, and component 2 (vertical) accounting for another 16.1%. NGT and T2DM samples were separated from each other along the primary axis in the analysis. Using a cut-off of 1% abundance, the main OTUs associated with the community metabolome along the first axis were those assigned to *Clostridiales*, *Subdoligranulum*, *Lachnospiraceae*, and *Roseburia* (Figure 3). *Roseburia* are known to produce butyrate, while *Clostridiales*, *Subdoligranulum* and *Lachnospiraceae* are opportunistic pathogens.

#### Correlations between clinical parameters and microbiota diversity

**Figure 4:**
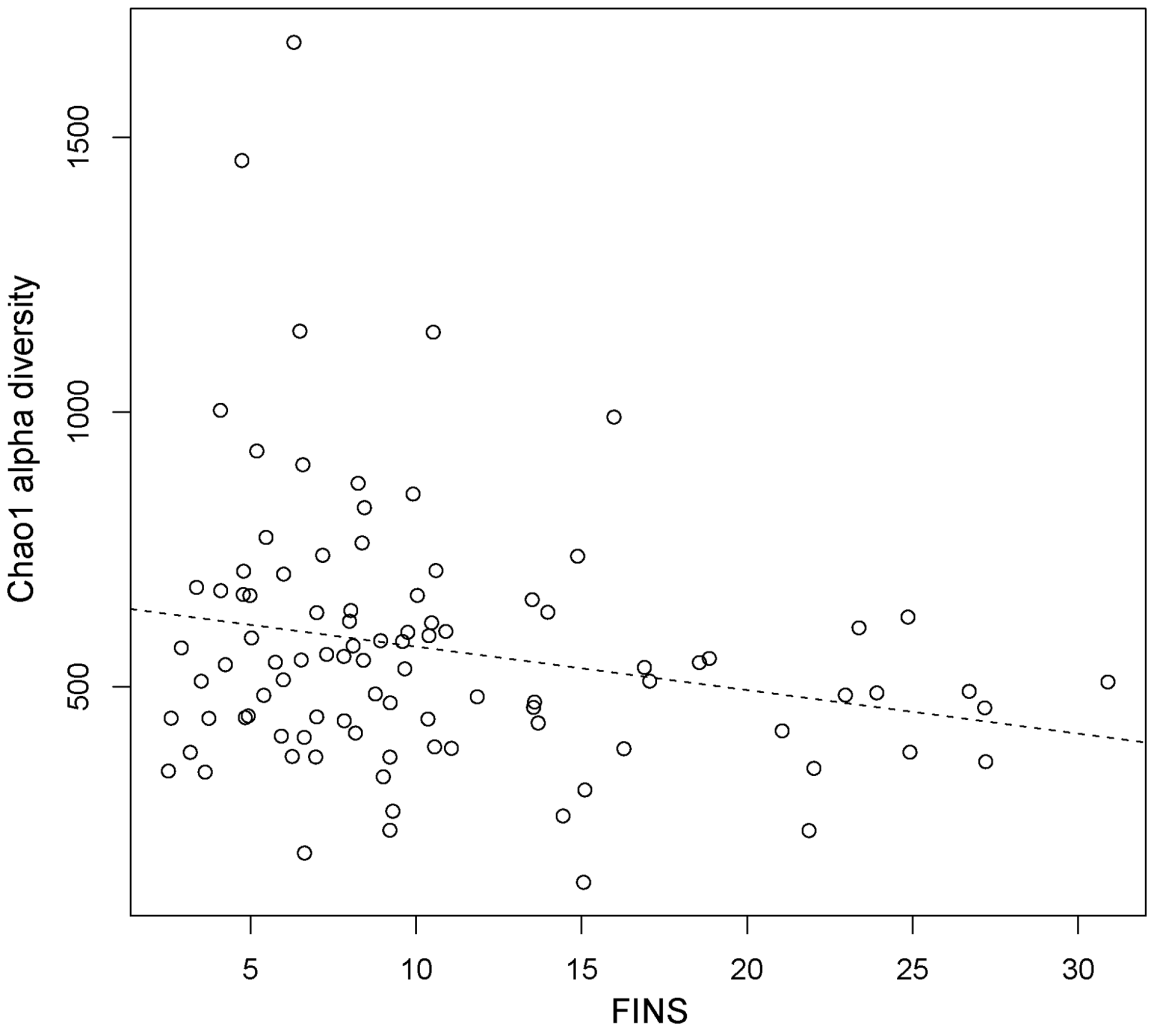
Correlations between the fasting insulin (FINS) concentration (mIU/L) and microbiota diversity. The dashed line was derived from a simple regression model (*P*-value for the model was 0.034; R = −0.22).

Stepwise multiple regression was conducted to test the relationship between clinical parameters and alpha diversity (Table S2). It was found that the FINS concentration was an independent relevant factor and was negatively correlated with the Chao1 index, which indicates that an insulin-resistant status has a relationship with the diversity of gut microbiota. Simple regression between the FINS and concentration and Chao1 diversity is shown in Figure 4.

### Validation and identification of T2DM markers

Some clades were identified as potential biomarkers for the diagnosis and management of T2DM by Kruskal-Wallis H tests based on changes in the relative abundance of each clade (Tables S3 and S4). In comparison with the NGT group, the relative abundance of *Verrucomicrobia* and Verrucomicrobiae were significantly decreased in the Pre-DM group. This indicates that Verrucomicrobiae can serve as a signal or a diagnostic biomarker for the progression of glucose intolerance, or it may even be a potentially beneficial microbe for the prevention of T2DM. Inversely, the relative abundance of Betaproteobacteria was significantly increased in the Pre-DM group in comparison with the NGT group, and was even higher in the T2DM group, which may be an indication of a high risk of T2DM. The abundance of *Clostridia* was also relatively higher in the T2DM group than in Pre-DM subjects, which suggests that it could be useful in the diagnosis of T2DM (Figure S5).

We also checked and compared the T2DM marker taxa reported by Larsen et al. [14] with our data (Tables S3 and S4 and Figure S6). Compared with the NGT group, the proportion of phylum *Firmicutes* and class *Clostridia* were a little more abundant in the diabetic group (*P* value = 0.07), but the ratio of *Bacteroidetes* to *Firmicutes* was not correlated with the plasma glucose concentration. Although these findings were not consistent with the study of Larsen et al. [14], similar results were observed in our study for class Betaproteobacteria which was highly enriched in diabetic subjects compared with NGT subjects.

To investigate the T2DM marker species found in the previous metagenomic study [16], we constructed a T2DM marker species profile by BLASTN each OTU's representative reads against 16s rDNA gene (filtered at 97% identity) of the T2DM marker species. The abundance of each species for each sample was calculated and normalized for the corresponding sample's total tags. A Kruskal-Wallis H test was performed between the NGT and T2DM groups based on this normalized profile (Table 4). At a first error of 5%, only 4 species were found to be significantly different among the 3 groups. *Faecalibacterium prausnitzii* L2-6 and *Haemophilus parainfluenzae* T3T1 were more abundant in the NGT group than in theT2DM group, which was consistent with the result of Qin et al. [16]. The former taxon is thought to be a butyrate-producing bacteria and healthy for humans. *Akkermansia muciniphila* ATCCBAA-835 also had a higher abundance in the NGT group than the Pre-DM group, while *Clostridiales* sp. SS3/4 was less abundant in the NGT group than the Pre-DM and T2DM groups. The results for these 2 species were inconsistent with the findings of Qin et al. [16]. Other markers were not significant, which may have been due to the small sample size.

**Table 4:** T2DM markers in the study^a^.

Species	*P*-value	Relative abundance mean			Occurrence rate		
		NGT	Pre-DM	T2DM	NGT	Pre-DM	T2DM
*Akkermansiamuciniphila* ATCCBAA-835	0.025	9.17E-05	1.16E-05	1.72E-05	0.227	0.063	0.077
*Bacteroides* 20-3	0.142	0.003859	0.003822	0.001607	0.909	0.906	0.769
*Bacteroides intestinalis* DSM17393	0.434	0.008022	0.003493	0.004447	0.841	0.766	0.769
*Clostridiales* sp. SS3/4	0.007	0.000846	0.001873	0.001716	0.886	0.953	1.000
*Clostridium* HGF2	0.271	0.000423	0.000115	0.000198	0.455	0.359	0.462
*Clostridium* SS2-1	0.209	0.012028	0.011949	0.018385	0.977	0.984	1.000
*Clostridium bolteae* str16351	0.590	0.001972	0.001052	0.001313	0.795	0.922	1.000
*Clostridium symbiosum* WAL14673	0.547	0.00055	0.000904	0.000954	0.614	0.734	0.769
*Eggerthella lenta* DSM2243	0.750	0.000678	0.00043	0.000421	0.591	0.516	0.538
*Escherichia coli* ED1a	0.315	0.004086	0.022505	0.007521	0.841	0.859	0.769
*Eubacterium eligens* ATCC27750	0.813	0.006233	0.009685	0.004943	0.773	0.813	0.769
*Eubacterium rectale* ATCC33656	0.915	0.021439	0.016453	0.032689	0.841	0.922	0.846
*Faecalibacterium prausnitzii* L2-6	0.031	0.054632	0.074787	0.04992	0.977	1.000	1.000
*Haemophilus parainfluenzae* T3T1	0.011	0.00018	0.000319	3.30E-05	0.364	0.531	0.077
*Roseburia intestinalis* XB6B4	0.862	0.040707	0.044163	0.060625	0.909	1.000	1.000
*Roseburia inulinivorans* DSM16841	0.942	0.007569	0.008261	0.005562	0.818	0.875	0.923
*Clostridium ramosum* DSM1402	0.658	0.001806	0.000649	0.002932	0.523	0.516	0.308

a These markers were identified in the study of Qin et al. [16].

The data in this table show the average relative abundance and occurrence rates in the three groups.

## Discussion

Pre-DM is a key stage in the progression from normal glucose metabolism to diabetes. Our data have shown that the relative abundances of *Bacteroides* and Clostridium undergo marked undulations with the progression of glucose intolerance. The relative abundances of Verrucomicrobiae and Betaproteobacteria from NGT to Pre-DM and T2DM showed an opposite trend, while the relative abundance of *Streptococcus* continued to decrease from NGT to Pre-DM and then to T2DM. Thus, our results support the hypothesis that dysbiosis in gut microbiota occurs early before the prediabetes stage, which suggests that the proportion and diversity of microbiota can serve as potential makers for a high risk of diabetes. Therefore, retaining the balance of gut microbiota in the prediabetic stage may be a unique intervention to delay the development of diabetes.

It was, however, surprising to find an inconsistency with regard to the enrichment of *Akkermansia muciniphila* when we compared data derived from this study using 16S rDNA gene amplicon sequencing with that derived from metagenomics [16]. There may be many reasons for this. Firstly, different target genes were used to calculate the abundances. 16S rDNA gene is a ubiquitous and extraordinarily conservative biomarker in terms of the key role it plays in the translational apparatus, and rare horizontal gene transfer has been reported [18]. Not all other genes are suitable for taxonomic assignment and therefore for abundance determination, especially as only a few genes were detected for some species. This study was based on 16S rDNA amplicon sequencing as it is theoretically representative of the microbial community structure. A better method for microbial characterization in metagenomics may be the clade-specific marker gene identified by Segata et al. [19]. This indicates the need for a combination of biomarker genes (amplicon sequencing and qPCR) and metagenomic shotgun sequencing.

Secondly, many other confounding factors such as dietary or pharmaceutical treatments may also affect gut flora [20], [21]. In the present study, no subjects were taking antibiotics, hormones, or other drugs such as glucose- or lipid-lowering agents so they could not significantly change the gut microbiota composition. Dietary factors are complex, and we did not quantitate each subject's dietary composition as is usually done in animal models. It has frequently been reported that the fat and fiber content of diets can affect gut microbiota composition [22]–[24]. As no dietary information was provided in the study of Qin et al. [16], a further study is needed to explore this.

As a mucin-degrading bacterium, *Akkermansia muciniphila* has been widely identified in the intestinal tract and considerable efforts have been made to explore its relationship with human health [25]. Recently, more and more reports have pointed out that this species plays a key role in maintaining the integrity of the mucin layer and reducing inflammation, and it also protects against obesity and T2DM via the same mechanism [26]. The strain *Akkermansia muciniphila* ATCCBAA-835 used by Everard et al. [26] was also identified in the present study and a higher abundance of this organism was observed in the NGT group. In addition, a negative correlation between *A. muciniphila* and body weight, fat mass and metabolic disorders associated with obesity has been observed [26]–[29]. Taken together, these findings and the decrease of Verrucomicrobiae in pre-DM indicate that the abundance of Verrucomicrobiae is meaningful for the diagnosis and risk estimation of T2DM. A similar conclusion was also reached by Liou et al. [30].

Interestingly, a higher abundance of genus *Collinsella* in T2DM compared with NGT and pre-DM was detected, and it has been reported that this genus shows a positive correlation with serum cholesterol, and is also enriched in patients with symptomatic atherosclerosis [31], [32]. This may indicate an association between *Collinsella*, T2DM and atherosclerosis.

Our data showed that among the 28 OTUs enriched in the NGT and T2DM groups, OTUs identified as *Haemophilus*, *Roseburia* and *Megamonas* were enriched in the NGT group, while OTUs identified as Lachnospiraceae, *Clostridiales*, *Ruminococcus*, *Eubacterium*, *Sporobacter*, *Abiotrophia*, *Peptostreptococcus* and *Subdoligranulum* had relatively high abundances in the T2DM group. It has been reported that almost all species of *Roseburia* are buryrate-producing bacteria and some species of *Megamonas* can ferment glucose into acetate and propionate [33], [34], and that all of these short-chain fatty acids (SCFAs) are beneficial for health. These findings indicate that T2DM is associated with compositional changes in the gut microbiota, especially a decrease of probiotics. Some of our findings are consistent with study of Larsen et al. [14] in 36 male adults, 18 of whom had T2DM. This study also found and validated the T2DM-associated microbiota markers that were identified in previous metagenome-wide study [16]. Some of these were opportunistic pathogens such as *Clostridiales*, *Subdoligranulum* and *Lachnospiraceae*. *Roseburia* which are known to be butyrate-producing bacteria, seem to have a protective role, as has been recognized in previous reports [35], [36]. The finding that *Roseburia* (OTU1900) had a notably high relative abundance in NGT subjects compared with the other study groups was in agreement with previously reported findings.

Growing evidence from animal studies has suggested that changes in gut microbiota composition in response to a high-fat diet results in endotoxemia and inflammation, which in turn triggers the development of obesity, insulin resistance and even diabetes [11], [37], [38]. Furet et al. [39] analyzed the gut microbiota composition in 13 lean individuals and 30 obese subjects, including 7 T2DM patients who underwent a Roux-en-Y gastric bypass. They found that there was a negative correlation between *F. prausnitzii* and insulin resistance status, and that the proportion of *F. prausnitzii* was directly linked to the reduction of low-grade inflammation upon therapeutic intervention in T2DM. *F. prausnitzii* was one of the 2 major butyrate-producing bacterial groups and a lower abundance was detected in pre-DM compared with NGT. Using quantitative real-time PCR, Fujimoto et al. [40] found an inverse correlation between the abundance of *F. prausnitzii* and disease activity in a study of 47 Japanese patients with Crohn's disease and 20 healthy subjects, and this decrease was independent of genetic background. As fermentative organisms, *F. prausnitzii and Roseburia* and *Eubacterium* spp. are thought to supply butyrate and other SCFAs to the colonic epithelium, which could reduce oxidative stress and inflammation [41]. The observation that inflammation occurs in association with a decrease of these benefical microbes, especially *F. prausnitzii*, indicates that this species plays key role in maintaining colonic epithelial cells.

According to the Meta-HIT study [17], individual gut microbiota can be assigned to 1 of 3 enterotypes driven by *Bacteroides*, *Prevotella* and *Ruminococcus*, respectively. In agreement with this, all 3 enterotypes were also observed in our study. No significant relationship was found between enterotypes and Pre-DM or T2DM status which was consistent with the results of a previous study [16].

In the present study, after adjustment for BMI, age and sex (which could be confounding factors), PERMANOVA tests showed that FPG and CRP concentrations were significantly associated with the gut microbiota composition. Another study in overweight adults found significantly lower concentrations of CRP and changes in gut flora composition after treatment with galacto-oligosaccharides, prebiotics that can positively modify the gut microbiota and immune system. The different clades included increased *Bifidobacteria* and decreased *Bacteroides* spp. and *C. histolyticum* [42]. This may be due to an increase in CRP in response to inflammation caused by bacterial infection or other conditions, and their role is to bind to phosphocholine on microbes and clear necrotic and apoptotic cells. Thus, in healthy subjects when gut flora are modulated by prebiotics such as galacto-oligosaccharides and fructo-oligosaccharides, beneficial microbes (including butyrate-producing bacteria and *Bifidobacteria* spp.) will become more abundant or have higher activity levels, and help to maintain epithelial cells and prevent inflammation and destruction of the mucus layer – which is important for normal intestinal tract function. This was confirmed by Lapthorne et al. [43] who found that inflammation is a consequence of the dysbiosis of gut microbiota. Interestingly, stepwise multiple regression showed that the FINS concentration was an independent relevant factor and was negatively correlated with the diversity of gut microbiota. Furthermore, in comparison with subjects with NGT, the FPG and FINS concentration and IR index were significantly higher in the Pre-DM group. These data are in line with the hypothesis that the structure of gut microbiota may have a relationship with the inflammation system and with an insulin-resistant status.

Over last 20 years, a series of diabetes prevention trials have indicated that lifestyle improvement, weight reduction, and medications such as metformin, acarbose and rosiglitazone can significantly reduce the risk of progression from prediabetes to diabetes and improve risk factors for cardiovascular disease [44]–[49]. However, most of the controlled clinical trials that have been conducted have focused on changes in glycemia as the primary outcome. Accumulating evidence indicates that the gut microbiota is involved in host metabolism by increased energy extraction, immune system modulation, and altered lipid metabolism, all which have been demonstrated to contribute to progression to T2DM [13], [50]–[52]. The association of gut microbiota and prediabetes not only provides a clue to monitor the natural course of diabetes, but also raises the possibility of manipulating the microbiotic environment to prevent or treat the onset of T2DM.

In conclusion, this is the first study to show that the proportion and diversity of intestinal microbiota are changed early in the period of prediabetes. Although the relatively low number of subjects was a limitation of the study, these findings enhance our understanding of the influence of changes in human gut microbiota on the pathogenesis of diabetes. Additional research is needed to explore the biologic plausibility and the dynamic interrelation between gut microbiota and diabetes.

## Design and Methods

### Subjects

A total of 121 adult subjects were enrolled in the study in Beijing, China and were divided into 3 groups according to WHO criteria for the diagnosis of diabetes [53]. The 3 groups were those with normal glucose tolerance (NGT group; n = 44, M/F ratio = 12/32, mean age 55±9 years); prediabetes subjects including those with impaired fasting glucose and impaired glucose tolerance (Pre-DM group; n = 64, M/F ratio = 23/41, mean age 54±7 years), and subjects with newly diagnosed T2DM (T2DM group; n = 13, M/F ratio = 7/6, mean age 52±9 years). Exclusion criteria were clinical existence of renal and/or hepatic diseases, gastrointestinal tract diseases, psychiatric disorders, neoplasia, and any other disease that could interfere with the conduct of the study. None of the participants had taken pharmacological doses of antibiotics, hormones, antiobesity agents, lipid-lowering drugs, antihyperglycemic agents, and probiotics at least 1 month before the study. The study complied with the principles expressed in the Declaration of Helsinki and was approved by the Ethics Committee of Peking University People's Hospital. Written informed consent was obtained from each participant of the study.

### Sample collection and processing

Venous blood samples were taken from study participants after an overnight fast for at least 10 hours, and serum samples were obtained in the normal manner. An oral glucose tolerance test (OGTT) was performed in each participant by measuring plasma glucose concentrations at 0 and 2 hours after oral ingestion of 75 g glucose. Aliquots of serum samples after fasting and 2 hours after glucose challenge were snap frozen in liquid nitrogen, and stored at −80°C until analyzed. Two fecal samples were also collected from each participant using sterile cups after defecation. One sample was used to perform routine tests to exclude subjects with bloody stools, pus or parasitic infections. Another fecal sample was brought to the laboratory within 2 hours and stored at −80°C for microbiota analysis.

### Clinical chemistry measurements

Plasma glucose concentrations were measured by the glucose oxidase method. Serum total cholesterol (TC), low-density lipoprotein cholesterol (LDL-C), high-density lipoprotein cholesterol (HDL-C), triglycerides (TG), C-reactive protein (CRP), and liver function and renal function indicators were measured using an automatic biochemical analyzer. Fasting insulin (FINS) and 2-hour insulin (2HINS) concentrations were determined by an electrochemiluminescence immunoassay (Elecsys 2010 system, Roche Diagnostics Ltd, Basel, Switzerland). Insulin resistance index (IR) was calculated from the fasting plasma glucose (FPG, mmol/L) and insulin (FINS, µU/ml) concentrations as: homeostasis model of assessment–insulin resistance (HOMA-IR) = FPG • FINS/22.5 [54].

### DNA extraction and pyrosequencing

DNA extraction and PCR amplification were performed as described previously [55]. The V3–V5 region of the 16S ribosomal RNA (rRNA) gene from each DNA sample was amplified using the bacterial universal forward primer 341F (5′-CCTACGGGNGGCWGCAG-3′) and the reverse primer 926R (5′- CCGTCAATTCMTTTRAGT-3′), with the following parameters: initial denaturation at 94°C for 3 min, followed by 30 cycles of 94°C for 45 s, 56°C for 1 min, and 72°C for 1 min with a final extension at 72°C for 10 min. Polymerase chain reaction (PCR) products were run on 1.5% agarose gel electrophoresis and the DNA band with the correct size was excised and purified using Wizard® SV Gel and PCR Clean-Up System (Promega, St. Louis, MO, USA). Sample-unique 10-base barcodes were added to the 5′ end of the primer 341F and used for sorting of PCR amplicons into different samples. Emulsion PCR was set up according to Roche's protocols for the three methods. Sequencing was performed on a 454 GS FLX titanium pyrosequencer (454 Life Sciences, Branford, CT, USA) at BGI-Shenzhen. All sequences used in this study are available from GenBank, Accession No. SRA068175.

### Bioinformatics analysis

All reads were filtered and trimmed as outlined by Pat Schloss using Mothur (v.1.21.1). Briefly, all reads shorter than 200 bp or with more than 8 homopolymers were removed. Two mismatches and no mismatches were allowed for the primer and barcode, respectively. The PyroNoise algorithm in Mothur (v.1.21.1) was implemented to remove noise from pyrosequenced amplicons with the default parameter [56]. Subsequently, all sequences were clustered using the program CD-HIT and both the similarity and the coverage for shorter sequences were set at 99% [57]. To determine the taxa for the samples at the OTU level, the most abundant sequence of each cluster generated by CD-HIT was selected as a representative and used to cluster into the OTU using QIIME with default parameters [58]. Each OTU's most abundant sequence was selected to determine the phylogeny of the OTU using the RDP classfier [59]. A taxonomic profile was generated based on the OTU annotation using PERL script.

### Statistical analysis

Clinical data were expressed as means ± standard deviation (SD) or medians (interquartile range), and differences between groups were compared by ANOVA (one-way analysis of variance). Relative abundances of gut microbiota were compared between the NGT, Pre-DM, and T2DM groups using the Kruskal-Wallis rank sum test. Richness and diversity estimations used the Shannon diversity and Chao1 index. Principal component analysis (PCA) was used to analyze the beta diversity between the 3 groups. PERMANOVA (permutational multivariate analysis of variance), Pearson correlations, and co-inertia analysis (CIA) were used to test the relationship between microbiota and clinical parameters.

## Supporting Information

Figure S1 **Relative abundance boxplot (121 samples) of the 20 most abundant clades.**
(TIF)

Figure S2 **Principal component analysis (PCA) results for the genus profile.**
(TIF)

Figure S3 **Enterotypes of the human gut microbiome.** Principal component analysis (PCA) figures were generated for the genus profiles. The 3 enterotypes [determined by the method of Arumugam et al. [17]] were labeled and grouped in the PCA figures. Fisher's exact test for the association between T2DM status and enterotypes was performed and was not significant (*P* = 0.3546).
(TIF)

Figure S4 **Changes in abundance for operational taxonomic units (OTUs,) that exhibited a significant difference in the Wilcoxon rank sum test between the NGT, Pre-DM, and T2DM groups.** All OTUs found to be changed significantly are labeled in different colors. The annotation for each OTU is listed in the corresponding color. The vertical arrows indicate that the corresponding OTUs have a higher or lower abundance.
(TIF)

Figure S5 **Average abundances of each clade in the 3 groups.** Taxons less than 1% and all tags assigned no genus in the 3 groups were counted as others. An average of 27.82%, 24.73% and 27.50% of the total tags could not be assigned to any known genus for the NGT, pre-DM and T2DM groups, respectively.
(TIF)

Figure S6 **Relationship between FBG (fasting blood glucose), PBG (postprandial blood glucose) and the ratio of** ***Bacteroidetes*** **to** ***Firmicutes*** **.** The lines in the figures were derived from a simple regression model (the model was not significant at alpha 0.05).
(TIF)

Table S1 **PERMANOVA test of the significance of the shift in gut microbiota structure.**
(DOCX)

Table S2 **Correlations between the Chao1 alpha diversity index and healthy parameters.** Stepwise regression was performed between the sample Chao1 alpha diversity index and healthy parameters by the “both” stepwise method. The final model Akaike information criterion (AIC) value was 1039.48, R2 value was 0.1119, and the *P*-value was 0.02898. The final model included SBP, FINS, TBIL, and Cr but only FINS was significant at the *P*<0.05 level. This result is consistent with the PERMANOVA test result, with every 8 mIU/L increase in FINS resulting in a 1 point decrease in the Chao1 alpha diversity index. *P<0.05; **P<0.001.
(DOCX)

Table S3 **Phylum level Kruskal-Wallis test.** ^a^The *Firmicutes* result was not significant at a first error rate of 5%. ^b^The *Verrucomicrobia* result was significant.
(DOCX)

Table S4 **Class level test.** Relative abundance mean = average relative abundance of each group; occurrence rate = ratio of samples that detected corresponding clades for each group.
(DOCX)
