# Supplementary figures and images for: Human Gut Microbiota Changes Reveal the Progression of Glucose Intolerance
Source: PLoS One. 2013 Aug 27;8(8):e71108. doi: 10.1371/journal.pone.0071108 (PMC3754967; doi:10.1371/journal.pone.0071108)

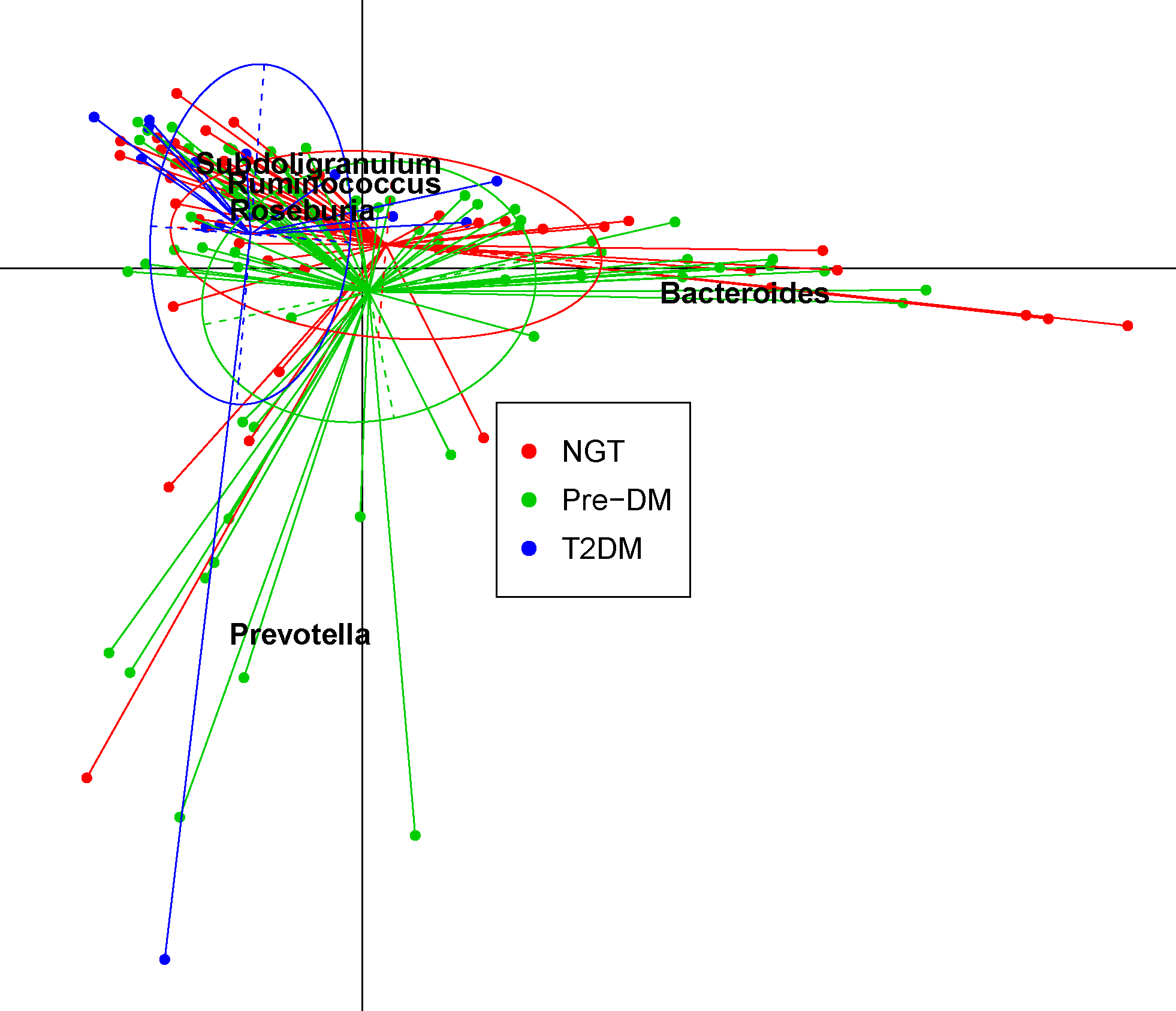

Supplement: Figure S2 — Principal component analysis (PCA) results for the genus profile. (TIF) [file pone.0071108.s002.tif]

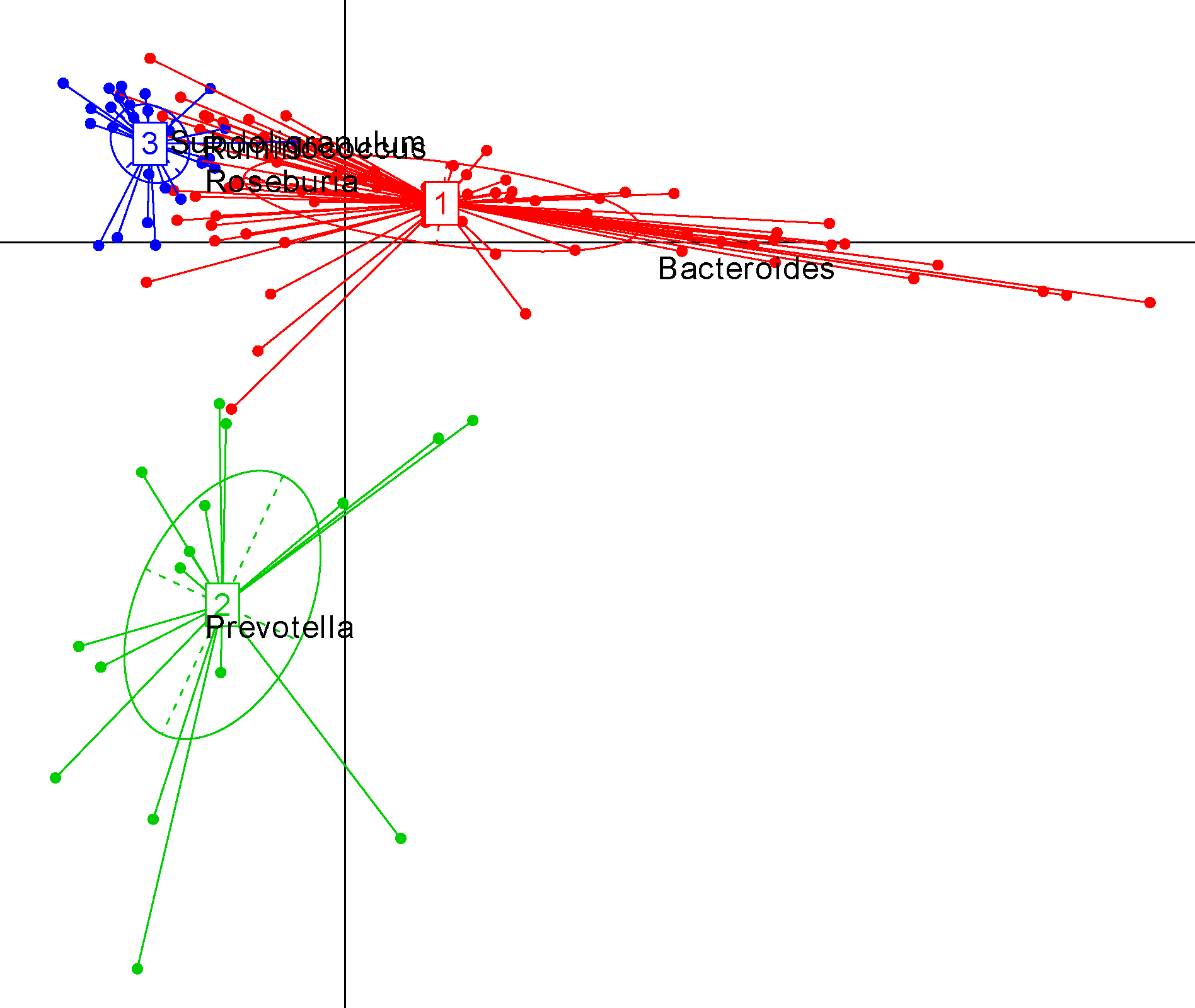

Supplement: Figure S3 — Enterotypes of the human gut microbiome. Principal component analysis (PCA) figures were generated for the genus profiles. The 3 enterotypes [determined by the method of Arumugam et al. [17]] were labeled and grouped in the PCA figures. Fisher's exact test for the association between T2DM status and enterotypes was performed and was not significant (P = 0.3546). (TIF) [file pone.0071108.s003.tif]

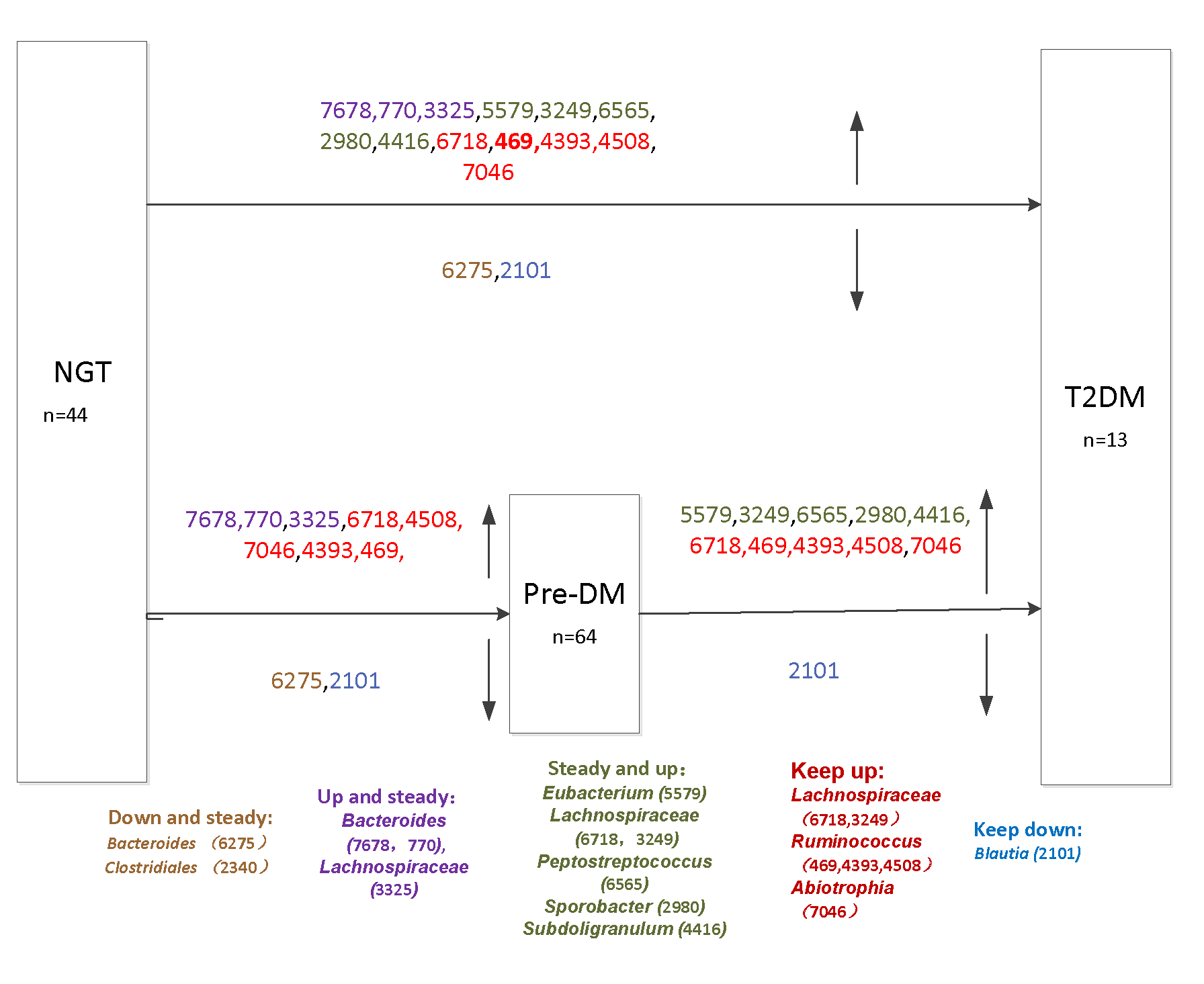

Supplement: Figure S4 — Changes in abundance for operational taxonomic units (OTUs,) that exhibited a significant difference in the Wilcoxon rank sum test between the NGT, Pre-DM, and T2DM groups. All OTUs found to be changed significantly are labeled in different colors. The annotation for each OTU is listed in the corresponding color. The vertical arrows indicate that the corresponding OTUs have a higher or lower abundance. (TIF) [file pone.0071108.s004.tif]

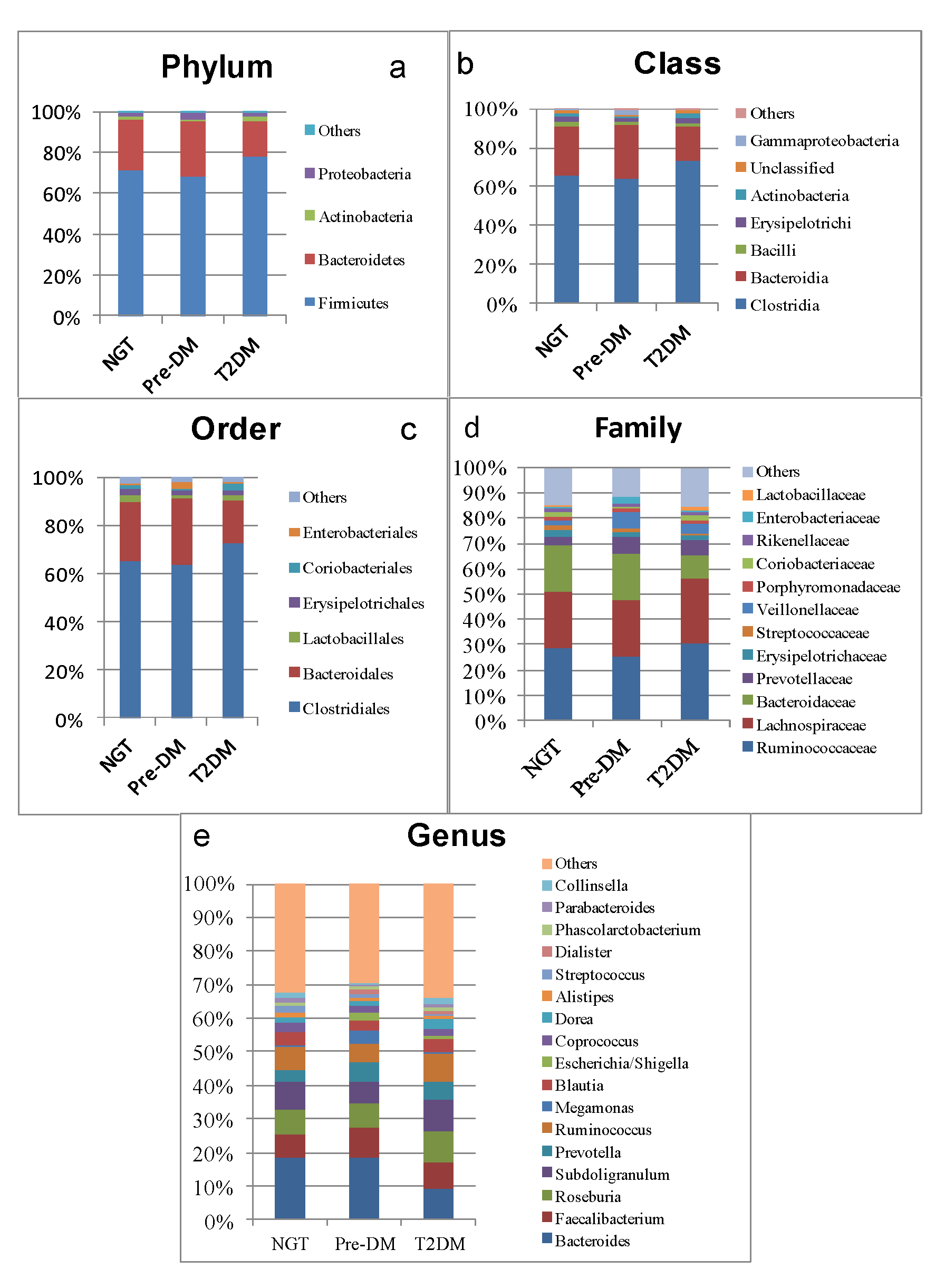

Supplement: Figure S5 — Average abundances of each clade in the 3 groups. Taxons less than 1% and all tags assigned no genus in the 3 groups were counted as others. An average of 27.82%, 24.73% and 27.50% of the total tags could not be assigned to any known genus for the NGT, pre-DM and T2DM groups, respectively. (TIF) [file pone.0071108.s005.tif]

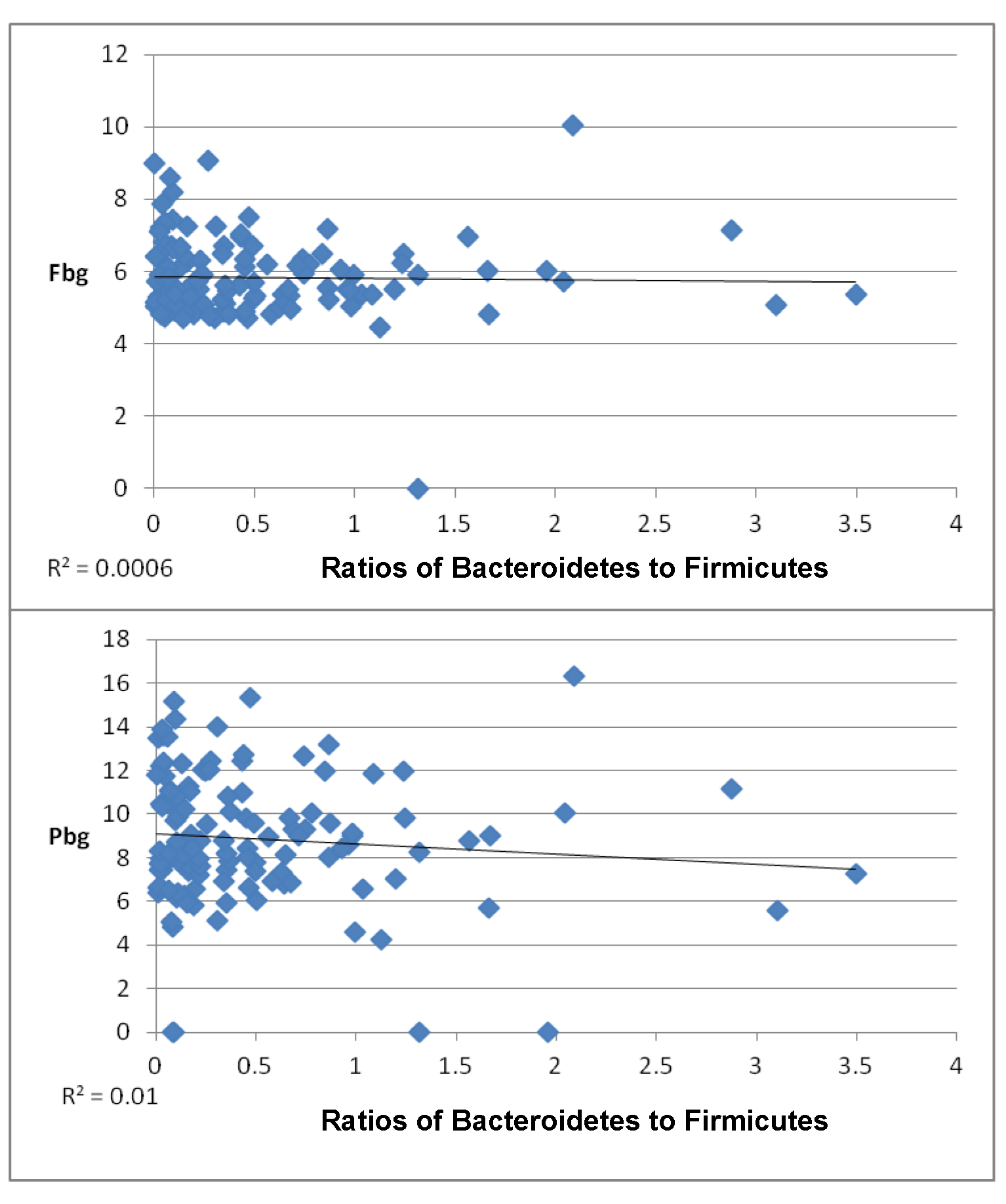

Supplement: Figure S6 — Relationship between FBG (fasting blood glucose), PBG (postprandial blood glucose) and the ratio of Bacteroidetes to Firmicutes . The lines in the figures were derived from a simple regression model (the model was not significant at alpha 0.05). (TIF) [file pone.0071108.s006.tif]
