# Supplementary material for: Human Gut Microbiota Changes Reveal the Progression of Glucose Intolerance
Source: PLoS One. 2013 Aug 27;8(8):e71108. doi: 10.1371/journal.pone.0071108 (PMC3754967; doi:10.1371/journal.pone.0071108)
Supplement: Table S1 — PERMANOVA test of the significance of the shift in gut microbiota structure. (DOCX) [file pone.0071108.s007.docx]

| **Table S1**. PERMANOVA test of the significance of the shift in gut microbiota structure | | | | | | |
| --- | --- | --- | --- | --- | --- | --- |
| Phenotype | Df | Sum of squares | Mean squares | F model | R^2^ | Pr (>F) |
| Type | 1 | 0.37873853 | 0.378739 | 1.100334 | 0.009162 | 0.2626 |
| PERMANOVA = permutational multivariate analysis of variance. | | | | | | |
