# Supplementary material for: Human Gut Microbiota Changes Reveal the Progression of Glucose Intolerance
Source: PLoS One. 2013 Aug 27;8(8):e71108. doi: 10.1371/journal.pone.0071108 (PMC3754967; doi:10.1371/journal.pone.0071108)
Supplement: Table S2 — Correlations between the Chao1 alpha diversity index and healthy parameters. Stepwise regression was performed between the sample Chao1 alpha diversity index and healthy parameters by the “both” stepwise method. The final model Akaike information criterion (AIC) value was 1039.48, R2 value was 0.1119, and the P-value was 0.02898. The final model included SBP, FINS, TBIL, and Cr but only FINS was significant at the P<0.05 level. This result is consistent with the PERMANOVA test result, with every 8 mIU/L increase in FINS resulting in a 1 point decrease in the Chao1 alpha diversity index. *P<0.05; **P<0.001. (DOCX) [file pone.0071108.s008.docx]

| **Table S2.** Correlations between the Chao1 alpha diversity index and healthy parameters | | | | |
| --- | --- | --- | --- | --- |
| Parameter | Estimate | Standard error | t-value | *P*-value |
| Intercept | 903.66 | 245.024 | 3.688 | 0.000386** |
| SBP | −2.76 | 1.727 | −1.599 | 0.1134 |
| FINS | −8.008 | 3.702 | −2.163 | 0.033181* |
| TBIL | −7.299 | 4.565 | −1.599 | 0.113394 |
| Cr | 2.961 | 1.855 | 1.596 | 0.113929 |
| Stepwise regression was performed between the sample Chao1 alpha diversity index and healthy parameters by the “both” stepwise method. The final model Akaike information criterion (AIC) value was 1039.48, R^2^ value was 0.1119, and the *P-*value was 0.02898. The final model included SBP, FINS, TBIL, and Cr but only FINS was significant at the *P* < 0.05 level. This result is consistent with the PERMANOVA test result, with every 8 mIU/L increase in FINS resulting in a 1 point decrease in the Chao1 alpha diversity index.  * *P* < 0.05; ** *P* < 0.001.  Cr = creatinine; FINS = fasting insulin concentration; SBP = systolic blood pressure; TBIL = total bilirubin. | | | | |
