# Supplementary material for: Human Gut Microbiota Changes Reveal the Progression of Glucose Intolerance
Source: PLoS One. 2013 Aug 27;8(8):e71108. doi: 10.1371/journal.pone.0071108 (PMC3754967; doi:10.1371/journal.pone.0071108)
Supplement: Table S3 — Phylum level Kruskal-Wallis test. aThe Firmicutes result was not significant at a first error rate of 5%. bThe Verrucomicrobia result was significant. (DOCX) [file pone.0071108.s009.docx]

| **Table S3.** Phylum level Kruskal-Wallis test | | | | | | | | | |
| --- | --- | --- | --- | --- | --- | --- | --- | --- | --- |
| Phylum | *P*-value | Relative abundance mean | | |  | | Occurrence rate | | |
|  |  | NGT | Pre-DM | T2DM | | NGT | | Pre-DM | T2DM |
| *Actinobacteria* | 0.159 | 0.016615 | 0.010984 | 0.015787 | | 0.977 | | 1.000 | 1.000 |
| *Bacteroidetes* | 0.104 | 0.251968 | 0.270251 | 0.163432 | | 1.000 | | 1.000 | 1.000 |
| *Proteobacteria* | 0.148 | 0.010857 | 0.02987 | 0.011722 | | 0.977 | | 0.984 | 1.000 |
| *Planctomycetes* | 0.417 | 2.91E-06 | 0 | 0 | | 0.023 | | 0.000 | 0.000 |
| *Synergistetes* | 0.284 | 2.66E-05 | 4.21E-05 | 7.17E-05 | | 0.136 | | 0.078 | 0.231 |
| *Firmicutes*^a^ | 0.091 | 0.712591 | 0.682742 | 0.801784 | | 1.000 | | 1.000 | 1.000 |
| *Cyanobacteria* | 0.376 | 3.24E-05 | 0.000201 | 0.000295 | | 0.159 | | 0.219 | 0.308 |
| TM7 | 0.417 | 1.52E-06 | 1.23E-06 | 6.36E-06 | | 0.023 | | 0.016 | 0.077 |
| *Deinococcus-Thermus* | 0.417 | 2.65E-06 | 0 | 0 | | 0.023 | | 0.000 | 0.000 |
| *Fusobacteria* | 0.314 | 3.20E-05 | 0.000657 | 7.36E-05 | | 0.136 | | 0.219 | 0.077 |
| *Euryarchaeota* | 0.507 | 1.00E-05 | 2.41E-06 | 0 | | 0.045 | | 0.016 | 0.000 |
| *Verrucomicrobia*^b^ | 0.025 | 9.17E-05 | 1.16E-05 | 1.72E-05 | | 0.227 | | 0.063 | 0.077 |
| ^a^ The *Firmicutes* result was not significant at a first error rate of 5%.  ^b^ The *Verrucomicrobia* result was significant.  NGT = normal glucose tolerance; Pre-DM = prediabetes; T2DM = type 2 diabetes mellitus. | | | | | | | | | |
