# Supplementary material for: Human Gut Microbiota Changes Reveal the Progression of Glucose Intolerance
Source: PLoS One. 2013 Aug 27;8(8):e71108. doi: 10.1371/journal.pone.0071108 (PMC3754967; doi:10.1371/journal.pone.0071108)
Supplement: Table S4 — Class level test. Relative abundance mean = average relative abundance of each group; occurrence rate = ratio of samples that detected corresponding clades for each group. (DOCX) [file pone.0071108.s010.docx]

| **Table S4.** Class level test | | | | | | | | | |
| --- | --- | --- | --- | --- | --- | --- | --- | --- | --- |
| Clade | *P-*value | Relative abundance mean | | |  | | Occurrence rate | | |
|  |  | NGT | Pre-DM | T2DM | | NGT | | Pre-DM | T2DM |
| Synergistia | 0.284195 | 2.66E-05 | 4.21E-05 | 7.17E-05 | | 0.136364 | | 0.078125 | 0.230769 |
| Alphaproteobacteria | 0.193717 | 0.000208 | 3.07E-05 | 0.000213 | | 0.204545 | | 0.15625 | 0.384615 |
| Methanobacteria | 0.506749 | 1.00E-05 | 2.41E-06 | 0 | | 0.045455 | | 0.015625 | 0 |
| Planctomycetacia | 0.416862 | 2.91E-06 | 0 | 0 | | 0.022727 | | 0 | 0 |
| Verrucomicrobiae | 0.025107 | 9.17E-05 | 1.16E-05 | 1.72E-05 | | 0.227273 | | 0.0625 | 0.076923 |
| Deltaproteobacteria | 0.612751 | 1.49E-05 | 1.07E-05 | 1.69E-05 | | 0.068182 | | 0.125 | 0.153846 |
| Deinococci | 0.416862 | 2.65E-06 | 0 | 0 | | 0.022727 | | 0 | 0 |
| Erysipelotrichi | 0.585759 | 0.022355 | 0.021373 | 0.025284 | | 1 | | 1 | 1 |
| Flavobacteria | 0.847765 | 2.67E-05 | 8.96E-06 | 0 | | 0.022727 | | 0.015625 | 0 |
| Sphingobacteria | 0.640624 | 0 | 3.58E-06 | 0 | | 0 | | 0.015625 | 0 |
| Cyanobacteria | 0.376191 | 3.24E-05 | 0.000201 | 0.000295 | | 0.159091 | | 0.21875 | 0.307692 |
| Bacteroidia | 0.1041 | 0.250889 | 0.270143 | 0.163362 | | 1 | | 1 | 1 |
| Betaproteobacteria | 0.025031 | 0.001509 | 0.003401 | 0.002958 | | 0.772727 | | 0.84375 | 1 |
| Fusobacteria | 0.314382 | 3.20E-05 | 0.000657 | 7.36E-05 | | 0.136364 | | 0.21875 | 0.076923 |
| Actinobacteria | 0.159431 | 0.016615 | 0.010984 | 0.015787 | | 0.977273 | | 1 | 1 |
| Clostridia | 0.074662 | 0.656395 | 0.64296 | 0.765691 | | 1 | | 1 | 1 |
| Ktedonobacteria | 0.416862 | 2.44E-06 | 0 | 0 | | 0.022727 | | 0 | 0 |
| Bacilli | 0.057819 | 0.028891 | 0.0164 | 0.008415 | | 1 | | 0.984375 | 0.923077 |
| Gamma-proteobacteria | 0.243041 | 0.009126 | 0.026427 | 0.008535 | | 0.931818 | | 0.96875 | 0.923077 |
| Relative abundance mean = average relative abundance of each group; Occurrence rate = ratio of samples that detected corresponding clades for each group. | | | | | | | | | |
